# Feasibility, acceptability, and appropriability of a national whole-school program for reducing school violence and improving school coexistence

**DOI:** 10.3389/fpsyg.2024.1395990

**Published:** 2024-06-24

**Authors:** Rodrigo Rojas-Andrade, Verónica Lopez Leiva, Jorge J. Varela, Pamela Soto García, Juan Pablo Álvarez, María Teresa Ramirez

**Affiliations:** ^1^Escuela de Psicología, Universidad de Santiago de Chile, Santiago, Chile; ^2^Centro De Investigación Para La Educación Inclusiva, Pontificia Universidad Católica De Valparaíso, Viña del Mar, Chile; ^3^Facultad de Psicología, Universidad del Desarrollo, Santiago, Chile; ^4^Observatorio de Género en Ciencias e Ingeniería, Universidad Técnica Federico Santa María, Viña del Mar, Chile; ^5^Ministerio de Educación de Chile, Santiago, Chile

**Keywords:** school violence, school climate, multilevel approach, feasibility, university technical assistance, whole-school approach

## Abstract

**Introduction:**

The increase in school violence following the COVID-19 pandemic underscores the need for schools to adopt a multilevel whole-school approach. This study examines a national program designed by the Chilean Ministry of Education, in collaboration with universities, as part of the Ministry’s Educational Reactivation Plan, aimed at improving school climate management across Chile.

**Methods:**

The “Learning to Live Together Program” (LLT) was implemented across all 16 regions of Chile, focusing on establishing school climate networks, providing direct intensive university technical assistance, and enhancing professional development and training. The feasibility, acceptability, and appropriability of the LLT program were assessed through a survey distributed to 1,561 staff members from 783 schools. Participants responded to a comprehensive set of instruments measuring acceptability, appropriability, feasibility, attitudes toward implementation, fidelity, and initial perceived results.

**Results:**

The results indicate high initial adoption rates and significant improvements in the assessed dimensions. The enhancement of school climate practices and strengthening school collaboration networks were of considerable relevance.

**Discussion:**

These findings support the efficacy of the multilevel whole-school approach as a viable strategy for Latin American countries, providing critical data for educational and governmental decision-making. Furthermore, this study provides evidence that these outcomes may be applicable to the implementation of similar policies in different contexts and countries.

## Introduction

After the COVID-19 pandemic, schools witnessed significant increases in reports of school climate and violence, including physical, verbal, and digital incidents among students, families, and teachers (López et al., 2022; Peist et al., 2024). In Chile, data from the Superintendence of Education (2024) show that the annual average of these reports rose from 8,872 during the pre-pandemic period (2014–2019) to 12,312 during the post-pandemic period (2022–2023), representing an increase of 38.76%.

This negative school climate is compromising learning environments and putting at risk the quality of educational systems (López et al., 2022; McMahon et al., 2022). To effectively address school violence, the emerging literature underscores the need for schools to adopt a whole-school approach (WSA) (Gaffney et al., 2021; Varela et al., 2021; Nyoni et al., 2022). Also known as “school-wide approaches” (Mayer et al., 2021) or “comprehensive school approaches” (Bradshaw et al., 2021), this approach calls for avoiding student-centered punitive and exclusionary approaches toward managing school violence (López et al., 2022) by placing the unit of intervention on the whole school, including teachers and school staff, providing positive, formative, preventive, and promotional strategies planned in multiple levels of the school unit, with varying degrees of depth and scope: whole-school positive activities for all children and classrooms; preventive strategies for some classrooms and children who need further support; and intensive one-to-one interventions for some students who still need further support. This approach is centered on the entire educational structure and its contents and is being gradually incorporated into educational policies at the global level (UNESCO, 2020; López et al., 2022).

WSA is based on Bronfenbrenner’s socioecological theory and considers school violence a multifactorial phenomenon that manifests through an interactive continuum of systems that influence the development of students and their social relationships. Therefore, WSA emphasizes the need to go beyond interventions centered exclusively on individuals involved in violent behaviors. These multi-tiered interventions can include the training and awareness-raising of students, teachers, families, and support staff, as well as the implementation of changes in school policies and practices (Hornby, 2016).

In addition, WSA interventions often have multiple components, incorporating various activities targeting all members of the school community. In some cases, they can also adopt a multi-level approach, offering different levels of prevention and intervention based on the specific needs of schools, groups, and individuals in three tiers: tier 1, universal interventions for everyone and all students; tier 2, group interventions for some students who show high mental health risk, frequent problematic behavior, or intermediate achievement levels in the development of socio-emotional skills; and tier 3, specialized interventions for only the few students who present social, emotional, or behavioral problems that require an intensive and personalized intervention (Ministerio de Salud, 2023).

### Implementation barriers for the whole school approach

Despite its wide dissemination, the success of the WSA has been limited (Valle et al., 2020). This is, to a great extent, because it is not recognized that schools are part of an education system and a community that is influenced by factors from an entire society (UNESCO, 2020). In Latin America, Chilean data has demonstrated that the management of school violence is not a priority and that efforts to tackle it are focused on the fulfillment of administrative obligations rather than on improving processes. According to Ascorra et al. (2021), this situation is related to the imbalance of the educational system, in which different school districts, especially public ones, have to respond to homogeneous standards from dissimilar base conditions, being forced to respond under bureaucratic accountability and with educational processes centered on high-stakes testing (López et al., 2021a,b).

Furthermore, the implementation of WSA faces a lack of definition in decentralized management models, ambiguity between the district coordinating roles, and lack of specialized training for professionals involved in these initiatives (Ascorra et al., 2021). In this scenario, López et al. (2022) conducted a multi-level analysis of 194 Chilean public schools from 65 school districts to examine how intermediate-level management could contribute to the implementation of WSA. The authors found that, instead of facilitating the adoption of WSA, school districts operate in a way that is more similar to barriers by placing resources and incentives in student-centered interventions, which are usually punitive and exclusionary for students´ educational trajectories. In this context, the contribution of the intermediate level to the progress of territories appears to be limited to the promotion of specific tier 3 interventions. In the absence of well-designed prevention and promotion strategies, these measures are excessively centered on individual students rather than prioritizing tier 1 and tier 2 as a strategy for the evidence-based improvement of school climate. The effect of the prioritization of tier 3 strategies is the individualization of school violence (López et al., 2011), which creates high stigmatization of students from contexts with more poverty, cultural diversity, and special educational needs (particularly ADHD), who receive more punitive exclusionary discipline measures (López et al., 2023).

In another study by Rojas-Andrade et al. (2023) on the organizational readiness of Chilean public schools for implementing WSA, a disconnect was found between school teams and teachers, with teachers claiming a perceived lack of support from their leaders and supervisors. In fact, the same study revealed that organizational readiness was not related to the technical training received by the teams, but rather to horizontal coordination and perceived social support between adult peers within schools.

These results are consistent with those of other international studies. Studying the implementation of WSA for the reduction of bullying in Iran, Salimi et al. (2021) found that imprecise school violence policies, lack of prioritization of this problem by school leaders and decision-makers, logistic difficulties, and funding limitations are highly critical obstacles. In addition, they identified that the presence of other issues in schools also acts as an obstacle when competing for resources and priority in the school agenda.

This same study underscored that the inclusion of multiple interested parties with different priorities and knowledge makes the cohesive implementation of an anti-bullying plan challenging (Salimi et al., 2021). In this scenario, Nyoni et al. (2022), in a systematic review of the WSA intervention to tackle gender violence, found that the lack of active involvement from families and members of the community can be a significant barrier, as it reduces the efficacy of intervention. This demonstrates that when families and the community are not adequately involved in the WSA, a vital component that can reinforce and support the changes initiated in the school environment is lost.

In line with the above, Ponsford et al. (2022), in a recent meta synthesis, indicate that although the implementation barriers of WSA can be summarized as the lack of understanding of the approach, weak commitment of the school staff, lack of continuous and reflexive collaboration between the school staff, restricted times, absence of effective leadership, and absence of a school culture that encourages innovation, all these obstacles are reduced when schools receive support to select, adapt, plan, and perform activities within the WSA. Pearce et al. (2022) believe that firm and clear policies, solid organizational structures, and systematic and continuous procedures over time contribute to improving the implementation climate of schools, increasing the organization’s readiness to face the challenges of the changes brought about by the WSA approach.

The scientific literature underscores the need for systems of robust support during the implementation of educational models, an aspect often undervalued in contemporary educational practices (López et al., 2022). This need is accentuated in decentralized school systems such as the Chilean system, in which the Constitution limits the capacity of the State to prescribe curricula or specific programs, such as anti-bullying initiatives or the promotion of a positive school climate. In this framework, the Ministry of Education of Chile (MINEDUC) has, since 2002, focused on providing guidelines through curriculum bases and national policies for school coexistence rather than specific instructions. Although this approach respects the freedom of teaching, it also poses challenges in terms of education uniformity and cohesion. The Education Quality Assurance System, established through Law N° 20.519, includes several institutions responsible for ensuring education quality, while MINEDUC plays a supervisory role. The other institutions—Superintendence of Education, Agency for Education Quality, and National Education Council—focus on inspection and evaluation. In this context, universities have emerged as key partners, complementing the role of MINEDUC through mediation in educational policies and the development of educational assessment programs (Pérez et al., 2013; Bellei et al., 2014; Carrasco-Aguilar et al., 2019). This highlights the importance of interinstitutional collaboration to strengthen the educational system.

Some promising experiences in Latin America resemble the whole school model by being comprehensive, systemic, and effective in the prevention of violence, such as the “Classrooms in Peace Multi-component Program” (in Spanish, “Programa Multi-componente Aulas en Paz”) of the research group Aggression, Conflicts and Education for Coexistence of Universidad de los Andes in Colombia. Inspired by the WSA, the program seeks to prevent aggression and promote peaceful coexistence through a curriculum for the development of citizen competences in the classroom, extracurricular efforts in groups of initially aggressive children who interact with children identified as prosocial, and workshops, visits, and phone calls to the families (Chaux, 2007; Ramos et al., 2007). The program combines tier 1 (citizen competences) and tier 2 strategies with components that may be feasible to implement in families and at the group level. In their results, a reduction in aggressive behavior, especially physical aggression, stands out, together with an increase in prosocial behaviors, particularly affection and care among classmates. In addition, a positive change was observed in the classroom, which in turn facilitated the realization of classes and other universal activities of the program. However, the application of this program in Chile did not show positive results (Ramos et al., 2007). In Chile, the “Paz Educa” program from Fundación Paz Ciudadana, which is also based on the WSA (Varela et al., 2009), evidenced positive results in the districts where it was applied in its first and subsequent implementations (Varela, 2011; Pérez et al., 2013). This program’s aim to improve coexistence and safety within educational establishments, positively impacting the organizational climate, enabling the teaching, and learning process, and the comprehensive development of children and adolescents. It is integral as it addresses interventions at different system levels: whole school, classroom, family, and individual; and preventative, as it targets all school students and not only those who present conflictive behaviors. The prevention levels were primary, secondary, and tertiary and were adapted from the public health literature on disease prevention. However, these experiences have not been translated or replicated in permanent support policies at the national level that allow for sustained change over time.

### University technical assistance as an implementation strategy for WSA programs

To overcome the barriers that limit the integration of evidence-based programs and policies in schools, advancing toward an implementation science approach is key. Implementation science is a field of study that focuses on researching how to adopt and effectively conduct evidence-based interventions in real-world scenarios. This approach focuses on understanding the factors influencing the successful implementation of practices, programs, and policies and seeks to develop strategies and approaches to maximize the adoption and impact of these interventions (Eccles and Mittman, 2006).

One distinction made by implementation science is between infrastructure and implementation systems. Wandersman et al. (2012), based on the *Interactive Systems Framework for Dissemination and Implementation*, proposed the existence of three systems that should work in alignment to achieve socially significant results. First, the Synthesis and Translation System distills the information and scientific evidence to translate it into usable innovations, recommendations, or programs. Second, the delivery system is responsible for the application and commissioning of innovation in organizations. Third, the support system aimed at supporting and facilitating implementation. The Support System assists implementation teams in identifying factors hindering and facilitating the correct and complete implementation of the intervention, proposing strategies for its optimization and reduction, assessing the results obtained by these strategies, and improving the intervention. As they are part of a system, their actions should be integrated into the intervention and work as a gear that ensures both fidelity to the design delivered by the synthesis system and sensitivity to the context in which the delivery system operates (Ovretveit and Tortolani, 2021).

Support system teams use implementation strategies (IE). IEs are methods or techniques employed to enhance the adoption, implementation, and sustainability of a specific program or practice (Powell et al., 2015). They are classified into “use evaluative and iterative strategies,” “adapt and tailor to context,” “develop stakeholder interrelationships,” “train and educate stakeholders,” “engage consumers,” “support professionals,” “utilize financial strategies,” “change infrastructure,” and “provide interactive assistance” (Waltz et al., 2015). One of the IEs most widely used and recommended worldwide is Technical Assistance (TA), which is applied in both the private and public sectors (Dunst et al., 2019; Scott et al., 2022).

TA is a personalized and practical method that supports the implementation of innovations and strengthens the skills of organizations and communities by generating solutions for specific challenges (Scott et al., 2022). Applied to the field of k-12 education, this strategy is designed to foster communication and enable school improvement processes that require organizational improvements through the creation of action communities and the promotion of effective strategies for achieving significant goals in the academic field and life of students (Katz and Wandersman, 2016; Maier, 2022). In this way, TA is conceptually consistent with the co-creation model that has recently become relevant to effective and sustainable implementation, as recipients of TA are active in the planning and implementation stages, which translates into contextualized practices that are adapted to the specific environment and emerge during the process (Metz and Albers, 2014; Yazejian et al., 2019).

Evidence indicates that the application of TA is highly efficient at both the individual and organizational levels (Scott et al., 2022). This facilitation strategy has been confirmed to significantly improve skills and knowledge and generate a positive impact on behavioral change. In addition, its use has been demonstrated to be closely related to a series of positive organizational results, such as successful implementation of programs, increased assessment capacity, improved quality of services, and collaboration among different groups. It should be noted that achieving sustainable improvements over time requires a higher sustained dose of TA and the commitment of both the leader and the organization’s staff to maintain these improvements over time (Dunst et al., 2019). Lastly, although different delivery modality and nuclear components of TA have been identified (Scott et al., 2022), the most relevant, according to Dunst et al. (2019) review, are needs assessment, co-definitions of goals and objectives, professional development and training, evaluation, and activities to increase the sustainability of activities.

### Technical assistance in education in Latin America

In Latin America, particularly in Chile, TA has been used since the 1990s, mainly by external teams such as supervisors from the Ministry of Education and private entities financed with public funds (Barrera et al., 2014). Local evidence indicates that on average, when at least 4 years of TA work accumulate, a positive and significant impact is achieved, which rapidly vanishes once the intervention is concluded if the capacities within the school are not effectively strengthened (González and Bellei, 2013).

Universities can operate as partners of governments in policy implementation and execution of TA programs, considering that in the global scenario, there is an increasing debate about improvement, initial training, and educational policies for TA professional development that neglect the critical and reflective role of universities (McIntyre et al., 2017; Brooks, 2021; Tillin, 2023). An example of successful experiences of University Technical Assistance is the National Implementation Research Network of the University of North Carolina in the United States, which undertakes the study and use of implementation science in diverse contexts. One of their projects is the State Implementation and Scaling-up of Evidence-based Practices (SISEP) Center funded by the Office of Special Education Programs of the U.S. Department of Education. SISEP helps states build infrastructure for the implementation and scaling-up of these practices. The TA provided by SISEP extends to state and local education agencies and engages in training district superintendents and management units. SISEP’s TA activities focus on stabilization, sustainability, scaling-up, and efficiency (Farmen et al., 2023).

Studies on TA conducted by the SISEP show that the support of state agencies is a decisive factor in achieving the expected results (Ward et al., 2022). The construction of this support depends widely on the expertise and knowledge of technical advisors, who should be experts in the development of implementation capacities in educational systems that are intrinsically variable and unique, adapting to the specifics of each context (Fixsen et al., 2009). Therefore, it is essential to understand how to implement TA interventions conducted by universities in unique educational systems such as those in Latin America, where an implementation science approach has been incorporated into the field of public policies.

### Objective of the study

The objective of this study was to analyze the feasibility of implementing a pilot university assistance program at the national level based on WSA for reducing violence and improving the school climate in public schools. This program was implemented by university teams and was led and coordinated by the Ministry of Education of Chile. This study has several relevant implications. First, it will address the results of a national WSA policy, providing evidence-based data that are crucial for decision-making at the government and education levels. In addition, the results of this study can serve as a model for the implementation of similar policies and programs in other contexts or countries. Finally, from a scientific perspective, this study significantly contributes to the existing literature, especially with respect to the development of effective implementation strategies on a national scale.

## Methods

### Learning to live together [“A convivir se aprende”] program

The Learning to Live Together Program (LLT) is one of the initiatives funded by the Ministry of Education (MINEDUC) of Chile, linked to the Reactivation Plan, which seeks to respond to schools’ needs and demands during the return to face-to-face classes after the COVID-19 pandemic.

LLT is a TA program that facilitates changes in the local education system. The approach consists of strengthening capacities in schools to integrate WSA to prevent school violence and improve school climate. The policy is linked to the National Policy for School Coexistence, an instrument that guides the work of schools on the subject. School coexistence is defined as the set of interactions and relationships among the members of a school. These interactions can create a harmonious and nurturing school climate that enhances teaching and learning processes and school engagement. On the other hand, when interactions are violent and characterized by the illegitimate use of force and power, they can result in physical or psychological harm to others, seriously affecting educational dynamics (DEG, 2019; MINEDUC, 2024). Thus, the LLT seeks to intervene and improve these aspects to ensure that schools can create inclusive and caring environments.

The LLT was designed to project its scaling-up to the national level through its implementation by universities located in different regions of Chile, with teams that specialize in school co-existence and mental health in collaboration with the professional teams of MINEDUC. The administrative-demographic application unity is the district, and all educational establishments (state and state-subsidized private schools) that receive public funding are invited to participate voluntarily. Each district participates for 2 years. The ACSA Program was designed as a multilevel model with three components:

**Universal TA** centered on school staff training: This component aims to disseminate and promote evidence-based practices aimed at creating inclusive and safe educational environments that foster the comprehensive development of students and enhance the knowledge and capacities of all schools in a city. **Two annual training encounters** are developed, which are aimed at management teams and school staff based on the diagnosis of local training needs. The ACSA program also developed a training course on the WSA in collaboration with the participating universities, creating audiovisual capsules about the WSA that are available to the public at https://convivenciaparaciudadania.mineduc.cl/escuela-total/.

Local **network TA** centered on building professional communities of practice. This component aims to implement a **school coexistence improvement network** among schools in the same district (Pávez, 2017) to strengthen the capacity of the local education system by promoting collaboration, knowledge exchange, and joint school initiatives. The regional and provincial teams of the Ministry of Education, which collaborate with the team of university advisors through **eight network meetings per year,** are responsible for the management and direction of this network.

**Intensive TA** focused on specific schools: This component focuses on offering intensive and specialized support for **ten sessions per year** for schools that require more TA according to the prioritization criterion of readiness for change combined with the psychosocial risk and school violence level defined by the provincial education teams. Intensive TA improves the plans and the quality of their implementation through onsite visits to schools. The number of schools to monitor is defined based on the size of the district (very small/small/medium/large), but the specific selection of schools is conducted by the Ministry of Education’s Provincial Department Office and district administrators (DAEM, Municipal Corporation, private administrators). After two years, prioritized schools stop receiving intensive TA.

As a public policy, the first year of the LLT program involved a pilot application in 60 of the 346 districts in the 16 regions of the country. In total, 17 universities participated. In each case, a team of advisors was formed, led by a researcher with an excellent track record. On average, each team comprised 12.05 (SD = 4.789) professionals, of whom most were psychologists (48.29%) and teachers (28.29%). These teams collaborate with professional teams from the Ministry in the territories, thereby enabling the development of educational policies based on scientific knowledge.

### Study design

The design is based on the design of feasibility studies and considers the following subdimensions: adoptability (feasibility, acceptability, appropriability); disposition and attitude toward implementation; and program fidelity. A survey design was applied before and after the launch of the program. During the first workshop session of Networking TA, the program was presented in detail through an audiovisual demonstration and a presentation of the expected activities and goals. Subsequently, the participants responded to an online survey that measured feasibility, acceptability, and appropriability. After the pilot program, the same survey was applied but complemented with a result perception instrument sent massively to the institutional e-mails of each participating school. Before responding, participants should have read and accepted the conditions for participation, which were established in an informed consent corresponding to the bioethical report No. BIOEPUCV-H-557-2022 by the Bioethics Committee of Pontificia Universidad Católica de Valparaíso.

### Participants

The first survey was responded to by 1,561 school staff members, corresponding to 783 educational establishments. The second survey was administered at the end of the first implementation semester and was completed by 548 people from 329 educational establishments. Table 1 shows the characteristics of the establishments that participated in the first survey.

**Table 1:** Characteristics of the establishments participating in the first survey (*n* = 783).

Variable	N	%
**Territorial zone**		
Center Macrozone	289	36.9%
South Macrozone	337	43.0%
Administrative dependency		
Delegated administration	3	0.4%
State	485	61.9%
Private	2	0.3%
State subsidized	203	25.9%
Local service of public education	90	11.5%
**Teaching level and modality**		
Pre-school education	399	51.0%
Primary education	618	78.9%
Secondary humanistic/scientific education	209	26.7%
Secondary technical/vocational education	137	17.5%
Art education	4	0.5%
Special education	47	6.0%
Adult education	42	5.4%
Size of management team		
10 or more members	77	9.8%
1–3 members	128	16.3%
4–6 members	395	50.4%
7–9 members	183	23.4%
**Years on the management team**		
0–4 years	486	62%
13 and more years	52	7%
5–8 years	196	25%
9–12 years	49	6%
**Management team members**		
Principal	744	95%
Coexistence head	703	90%
Technical chief	677	86%
School manager	500	64%
Head of education integration program	459	59%
Counselor	248	32%
Psychologist and social worker team	227	29%
Cycle coordinator	143	18%
Deputy principal	86	11%
Technical professional education head	76	10%
Department head	38	5%
Other (specify)	282	36%

### Instruments

#### Establishment characterization measures

*Ad hoc* measures were developed to describe the characteristics of the participating education establishments. These include: region, zone, district, position of the respondent, dependency of educational establishment, teaching level and modality, size, composition, and experience of the school co-existence management team.

Acceptability of Intervention Measure (AIM), Intervention Appropriation Measure (IAM), and Feasibility of Intervention Measure (FIM), developed by Weiner et al. (2017). These measures were adapted into Spanish and adjusted for LLT. Each dimension has a four-item structure, and its psychometric robustness has been demonstrated in previous studies. The highest scores reflect a higher adoptability of the intervention within the program. Owing to their simplicity, robustness, and utility, these measures are now being used in health (Makhtar et al., 2024) and education (Lawson et al., 2023). For this reason, their use is recommended for the study of school program implementation (Schultes, 2023).

#### Disposition and attitude toward implementation scale

The instrument comprised five questions on a 10-point Likert-type scale that assessed participants’ confidence in the program, its importance, readiness for implementation, perceived success, and trust in its efficiency. If any question receives a score below 8, two additional questions will be asked to identify possible actions that may increase acceptance and trust in the program. The application of this scale provides a full panorama of the disposition and attitude toward implementation, allowing for detecting areas of improvement to ensure implementation success.

#### Fidelity assessment scale

This instrument was developed to measure the effectiveness and perception of LTT implementation in education communities. It is based on the implementation fidelity multidimensional assessment model (Rojas-Andrade and Leiva, 2019) and assesses the following five dimensions: adherence to essential components, professional expertise, dose, receptiveness of participants, and results perception. Each dimension is assessed using various items on a 5-point Likert scale.

### Analysis

Data were analyzed by considering the school as the unit of analysis, and its database number (RBD) was used as the identifier. Individual responses were grouped around this identifier using median scores. This measure was selected because of its capacity to reduce the end point bias compared with the mean, especially when there is limited observation. In the first application, the average number of participants per establishment was 1.94 (MIN = 1; MAX = 10; SD = 1.27), whereas in the second application, it was 1.63 (MIN = 1; MAX = 7; SD = 0.994). For the descriptive statistics analysis, central tendency and dispersion measures were employed as interval variables. Despite the scales being ordinal, all of them had more than 5 response points, allowing for a parametric analysis. Therefore, we decided to work with percentage scores to increase interpretability; in this way, scores above 70% were considered high.

Significance tests were used to compare the scores between the characterization variables. To compare the pre- and post-implementation answers, data were matched through each school’s identifier using 273 cases. A segmented analysis based on educational establishment characteristics was not considered because it exceeds the scope and objectives of this study.

Finally, different regression models were conducted to explain the variation in the implementation results, using the territorial and organizational variables as criteria, as well as the implementation fidelity variables.

## Results

### Adoptability of the program

The initial adoptability was high. In the case of acceptability, a mean score of 4.33 (SD = 0.64; 87%) was found, while appropriability was 4.23 (SD = 0.62; 86%) and feasibility was 4.09 (SD = 0.59; 81.70%). When comparing the initial and end scores, all dimensions were found to have significant increases, as shown in Table 2.

**Table 2:** Comparison of the adoptability variables of the program before and after its implementation.

Variable	M before	SD before	M before	SD before	Difference	Standard deviation of the difference	*t*	gl	*p*
Acceptability	4.354	0.607	4.465	0.696	−0.110	0.804	−2.266	272	0.024*
Appropriability	4.308	0.545	4.401	0.672	−0.093	0.757	−2.030	272	0.043*
Feasibility	4.123	0.548	4.258	0.626	−0.135	0.706	−3.160	272	0.002**

**p < 0.001; *p < 0.05.

### Attitudes and disposition toward implementation

To assess the perception of the implementation of the program before and after its launching, five items were used, measured on a 10-point scale. These were: “How confidently can you explain what the program is about to other people?” (M = 5.90, SD = 1.68, 59%), “How important is it to implement the program in your educational community?” (M = 6.97, SD = 1.39; 70%). “How ready are you to implement the program in your educational community?” (M = 5.87, SD = 1.66; 59%), “How successful would the program be if it were implemented in your educational community tomorrow?” (M = 6.31, SD = 1.57; 63%), and “How confident are you that this program will work?” (M = 6.50, SD = 1.48; 65%).

The results reflect a moderate (<80%) or ambivalent perception (given the dispersion of the responses) about the implementation of the program. Therefore, although their importance is acknowledged and there are some expectations for success, the mean scores below 8 indicate some uncertainty and lack of complete conviction in the personal preparation, success, and functionality of the program in the educational communities before implementation.

When comparing the change of perception before and after the launch of the LLT Program, the results show increases in all assessed areas: confidence to explain what the program is about [*t*(259) = −9.886, *p* < 0.001], importance of implementing the program in the educational community [*t*(225) = −9.825, *p* < 0.001], preparation for the implementation of the program [*t*(260) = −9.170, *p* < 0.001], perceived success if the program were implemented tomorrow [*t*(253) = −6.957, *p* < 0.001], and confidence that the program will work [*t*(256) = −7.277, *p* < 0.001]. These findings show a positive trend in the perception of the assessed educational community in terms of understanding, importance, readiness, success, and confidence in the LLT program.

### Fidelity of implementation during the application of the LLT program

The general fidelity perception found in the program was 85%, which was considered high. As shown in Table 3, all fidelity indicators were positively valued. Regarding adherence, 84% was reached, with the pertinence of the topics being the most valued and the quality of materials the least valued, while dose obtained 81%. The most valued indicator was performance during the planned day, while performance at the right moment for school obtained the lowest score.

**Table 3:** Valuation of fidelity indicators of implementation of the program during launch.

Variables	Mean	SD
Adherence to the program		
Had clear objectives shared by the school members	4.203	0.782
Addressed topics that were pertinent for the needs and problems that we face as a school	4.364	0.689
Followed a logical sequence based on the whole school approach	4.275	0.704
Used methodologies comfortable for the school	4.220	0.762
Was adapted to the specific characteristics and conditions of the school and its members	4.148	0.766
Was complemented with useful and attractive materials	4.112	0.789
Dose		
Had an adequate number of sessions	3.894	0.870
Had an optimal duration	4.053	0.803
Was conducted at the right moment for the school	3.495	1.068
Started on time	4.143	0.830
Was performed on the planned days	4.412	0.692
The conditions of the space used were warm and comfortable	4.394	0.716
Receptiveness of participants		
Were committed to the activities	4.394	0.692
Knew the benefits of participating in the activities	4.179	0.785
Actively participated in the activities	4.383	0.721
Were happy with the activities performed in the workshop	4.311	0.745
Agreed on the problem and/or need that the activities aimed to solve	4.344	0.729
Professional expertise		
Managed the contents in an expert way	4.432	0.721
Managed the methodology of the activities in an expert way	4.405	0.717
Considered the opinions and concerns of the school members	4.480	0.711
Showed sufficient competences to apply the program	4.432	0.686
Have been responsible with their acquired commitments	4.526	0.686
Maintained a good relationship	4.689	0.614

Regarding the receptiveness of participants, 86% were reached. The most valued indicator was commitment to the activities performed, whereas the indicator with the lowest score was knowledge of the benefits of the program. Finally, 90% was obtained for professional expertise. The most valued dimension was the good treatment of professionals, whereas the least valued dimension was expertise in the methodologies used.

### Perception of the program’s results

Participants of the program perceived improving the practices of school coexistence as the best outcome, followed by strengthening the collaboration network between schools, as shown in Table 4.

**Table 4:** Valuation of indicators of the program’s results during its launch.

Indicators	Mean	SD
Know the reality of other educational establishments of my district	4.401	0.716
Generate/strengthen a collaboration network between schools that can last over time	4.302	0.736
Improve the school coexistence management practices	4.339	0.727
Incorporate a multilevel and whole school perspective into the plans of the coming year	4.225	0.784
Recognize the organizational resources that the school has to face the crisis	4.211	0.747
Identify the needs and resources of the territory/district to better address school coexistence management and crisis intervention	4.267	0.750

### Fidelity and adoptability

To analyze the possible explanations for the variations encountered in acceptability, appropriability, feasibility, and attitudes toward implementation before and after the launch of the program, multiple regression analyses were conducted, as shown in Table 5.

**Table 5:** Comparison of multiple regression models for pre-implementation variables.

Variable	Acceptability (B, SD)	Appropriability (B, SD)	Feasibility (B, SD)	Attitudes toward implementation (B, SD)
Constant	−2.141 (0.000)**	−2.825 (0.000)**	−1.684 (0.000)**	−0.398 (0.618)
Adherence	0.317 (0.016)*	0.171 (0.140)	0.272 (0.020)*	0.260 (0.383)
Dose	0.057 (0.645)	0.107 (0.333)	0.061 (0.581)	−0.067 (0.818)
Participant receptiveness	−0.069 (0.594)	0.308 (0.007)**	0.193 (0.095)	0.460 (0.113)
Professional expertise	0.232 (0.088)	0.215 (0.073)	0.010 (0.931)	−0.348 (0.233)
Perceived results	0.003 (0.981)	−0.138 (0.257)	−0.109 (0.376)	0.058 (0.863)
Administrative dependence	−0.009 (0.930)	0.113 (0.193)	0.111 (0.206)	−0.007 (0.972)
Team size	−0.009 (0.946)	−0.025 (0.826)	−0.092 (0.420)	−0.297 (0.344)
Years of experience	−0.055 (0.561)	0.000 (0.997)	−0.009 (0.911)	−0.045 (0.833)
North Macrozone	−0.033 (0.810)	0.039 (0.750)	−0.024 (0.846)	−0.006 (0.984)
Center Macrozone	−0.087 (0.387)	−0.036 (0.683)	−0.111 (0.216)	−0.003 (0.988)
*F*	5.526	9.829	4.565	0.903
*R* ^2^	−174	0.273	0.148	0.043

**p < 0.001; *p < 0.05.

Adherence exhibited a significant and positive relationship with acceptability (*B* = 0.317, *p* < 0.05) and feasibility (*B* = 0.272, *p* < 0.05), whereas the receptiveness of participants correlated with appropriability (*B* = 0.308, *p* < 0.01). The dose, professional expertise and perceived results variables did not show any significant association with any dependent variable, nor were significant associations found with administrative dependence, team size, years of experience and geographic zone where the program was implemented.

These findings indicate that both adherence and participant receptiveness play an important role in the acceptability and feasibility of the program. The model explains a moderate proportion of variability in acceptability (*R*^2^ = 0.174) and appropriability (*R*^2^ = 0.273), indicating that other factors outside this model may influence the variables.

## Discussion

The objective of this study was to analyze the feasibility of a University Technical Assistance pilot program at the national level conducted by university teams to implement a national WSA to mitigate violence and improve the school climate in public schools. The results show that the initial adoption of the program was high, indicating that participants were willing to implement it in their educational communities. In addition, all assessed dimensions (acceptability, appropriability and feasibility) experienced significant increases from the beginning of the program until its implementation.

One of the distinctive characteristics of the LLT program is the participation of education communities in the planning and implementation of the program. This promotes the creation of contextualized, specific, and emergent practices in each process (Metz and Albers, 2014; Yazejian et al., 2019). The co-creation of knowledge and suitable practices may lead to a more efficient adoption of innovations in the educational context (Alderman, 2018; Ley et al., 2022), which is fundamental to guarantee sustained change, as indicated by Lennox et al. (2020).

Regarding attitudes and disposition to conduct implementation, participants had a moderate or ambivalent perception before launching the program. Although they recognized its importance and had expectations for success, mean scores below 8 indicate some uncertainty or lack of complete confidence in personal readiness as well as the success and usefulness of the program. However, during the implementation of the program, significant increases were observed in all assessed areas of attitudes and dispositions. This demonstrates that despite the phenomenon known as voltage drop (Chambers et al., 2013) expected during the first period of the launching process, the TA offered by the university teams improves the program’s adoptability.

With respect to implementation fidelity during the application of the program, the general perception was high fidelity. Fidelity indicators were positively valued, and high adherence, dose, participant receptiveness, and professional expertise levels were achieved. This reveals that the program was implemented efficiently and successfully. Regarding the perception of the program’s results, participants perceived the improvement of school coexistence practices and strengthening of the collaboration network between schools as the most crucial outcomes. This is plausible considering that the implementation strategy employed allows for the personalization and reinforcement of skills in the communities (Scott et al., 2022). However, a long-term assessment is necessary to confirm these results. Additionally, to understand the efficacy of the program, more research is required to obtain evidence-based data about the program’s impact on school climate and violence reduction.

In the multiple regression analysis, some significant relationships were found between the variables. Adherence showed a positive relationship with acceptability and feasibility, whereas participant receptiveness was related to appropriability. However, dose, professional expertise, and perceived results did not yield significant associations. These data are relevant because implementation support in educational systems strongly depends on the expertise and capacities of technical advisors (Fixsen et al., 2009).

In summary, these results indicate high adoption and fidelity to the program and an improvement in attitudes and disposition to implementation during its launching. However, strengthening certainty and conviction before program implementation is necessary to ensure an even more significant impact.

At the same time, positive results might require at least 4 years of TA work to foster capacities within the educational community (González and Bellei, 2013). Therefore, the sustainability of the current impact is at risk if the implementation of the program is not extended. Consequently, the availability and resources necessary for the implementation and maintenance of the program in the long term should be discussed, as not guaranteeing financial and operative sustainability is a critical factor to ensure its continuity and impact in the long term.

Finally, it is noteworthy that this study has the following limitations. The results only allow for answering some questions about the universities “TA role.” For example, the professional competence of the execution times is highly valued; however, further qualitative research is needed to address other questions, such as the quality of the collaboration with MINEDUC. Another limitation is that the model focuses only on urban and scientific-humanistic schools, without considering the rural and technical-vocational context, which is also relevant due to the high student enrollment rate in the country and might also benefit from the whole school model.

## Conclusion

This study shows that the implementation of a technical assistance program to improve school climate and mitigate violence in public schools, with the characteristics of the ACSA Program design, may be successful in terms of initial adoption, fidelity, and improvement in attitudes and disposition to implementation.

Based on these results, the program envisions, with extensive possibilities, generating a positive impact on the change of behavior and positive organizational results of the educational communities. With these results at its disposal, the Government of Chile, through the Ministry of Education, committed to maintaining and increasing the coverage of the ACSA Program for the 2023–2025 period. In its second year, the program maintains its original design, improving some weaknesses identified in the present study. In this way, for example, from the second implementation year, there is audiovisual material to support the tier 1 training processes with capsules of specialized professors from some of the participating universities and transforming educational experiences at the establishment and intermediate levels. Therefore, the initial period of the program is improved on the basis of the year.

Despite the high efficacy of the program, some areas for improvement have been identified, such as strengthening conviction and certainty before program implementation, as well as considering the financial and operative sustainability in the long term to guarantee a continuous impact. Additionally, the need for further research to better understand the collaboration between universities and the Ministry of Education is also mentioned.

Furthermore, the program enables a support system for implementation that is common within educational models, which, despite not prescribing content, actively and directly helps transform practices through real integration into daily school life. This is a relevant milestone considering the difficulties that arise in a decentralized school system that is only allowed to provide guidelines. Finally, this study provides valuable information to adjust and improve future implementations of a university technical assistance program with national scope in educational communities in the Latin American region. Thus, the results of this study can serve as a model for the implementation of similar policies and programs in other contexts or countries, which can be scaled up by ministerial education entities in collaboration with the universities of their respective countries.

## Data availability statement

The raw data supporting the conclusions of this article will be made available by the authors, without undue reservation.

## Ethics statement

The studies involving humans were approved by bioethical report No. BIOEPUCV-H-557-2022 by the Bioethics Committee of Pontificia Universidad Católica de Valparaíso. The studies were conducted in accordance with the local legislation and institutional requirements. The participants provided their written informed consent to participate in this study.

## Author contributions

RR-A: Conceptualization, Data curation, Formal analysis, Investigation, Methodology, Resources, Software, Supervision, Validation, Visualization, Writing – original draft, Writing – review & editing. VL: Conceptualization, Data curation, Formal analysis, Investigation, Methodology, Validation, Writing – review & editing. JV: Formal analysis, Investigation, Methodology, Validation, Writing – review & editing. PS: Conceptualization, Investigation, Methodology, Validation, Writing – review & editing. JÁ: Resources, Supervision, Validation, Writing – review & editing. MR: Resources, Supervision, Validation, Writing – review & editing.
